# Careful treatment planning enables safe ablation of liver tumors adjacent to major blood vessels by percutaneous irreversible electroporation (IRE)

**DOI:** 10.1515/raon-2015-0031

**Published:** 2015-08-21

**Authors:** Bor Kos, Peter Voigt, Damijan Miklavcic, Michael Moche

**Affiliations:** 1 University of Ljubljana, Faculty of Electrical Engineering, Ljubljana, Slovenia; 2 Leipzig University Hospital, Department of Diagnostic and Interventional Radiology, Leipzig, Germany

**Keywords:** irreversible electroporation, liver tumors, colorectal carcinoma, finite element method

## Abstract

**Background:**

Irreversible electroporation (IRE) is a tissue ablation method, which relies on the phenomenon of electroporation. When cells are exposed to a sufficiently electric field, the plasma membrane is disrupted and cells undergo an apoptotic or necrotic cell death. Although heating effects are known IRE is considered as non-thermal ablation technique and is currently applied to treat tumors in locations where thermal ablation techniques are contraindicated.

**Materials and methods.:**

The manufacturer of the only commercially available pulse generator for IRE recommends a voltage-to-distance ratio of 1500 to 1700 V/cm for treating tumors in the liver. However, major blood vessels can influence the electric field distribution. We present a method for treatment planning of IRE which takes the influence of blood vessels on the electric field into account; this is illustrated on a treatment of 48-year-old patient with a metastasis near the remaining hepatic vein after a right side hemi-hepatectomy.

**Results:**

Output of the numerical treatment planning method shows that a 19.9 cm3 irreversible electroporation lesion was generated and the whole tumor was covered with at least 900 V/cm. This compares well with the volume of the hypodense lesion seen in contrast enhanced CT images taken after the IRE treatment. A significant temperature raise occurs near the electrodes. However, the hepatic vein remains open after the treatment without evidence of tumor recurrence after 6 months.

**Conclusions:**

Treatment planning using accurate computer models was recognized as important for electrochemotherapy and irreversible electroporation. An important finding of this study was, that the surface of the electrodes heat up significantly. Therefore the clinical user should generally avoid placing the electrodes less than 4 mm away from risk structures when following recommendations of the manufacturer.

## Introduction

Irreversible electroporation (IRE) is a tissue ablation method which relies on the phenomenon of electroporation.^1^ Electroporation occurs, when cells are exposed to sufficiently strong electric fields. These fields disrupt the plasma membrane and cause increased permeability of the plasma membrane to ions, larger molecules, and even DNA. When the electric field is sufficiently strong, the cells cannot recover from the disruption of the membrane and consequently undergo apoptotic or necrotic cell death.^2^,^3^ When this method is used for curative tumor ablation, it requires the whole tumor to be covered with sufficiently strong electric fields, which requires placement of at least two, but typically four or more electrodes around and/or inside the tumor. The electrodes can be positioned percutaneously under ultrasound or CT guidance, or intra-operatively.^4^,^5^

IRE relies on applying electric fields in excess of 600 V/cm in the target volume.^6^,^7^ Since tissues are rather good conductors, and tissue conductivity even increases during pulse application, electric fields in tissue are accompanied by significant currents, which can be up to 50 A (maximum current limit of the Nanoknife^®^ device). This leads to large power dissipation in the tissue during the pulses, which can be up to 150 kW during the pulse. However, the pulses are typically equal or less than 100 μs long and always delivered synchronized with the heart rate, which usually is lower than 100 beats per minute. This results in duty cycles of less than 0.1 % and consequently the actual delivered power is less than 15 W, which is between a factor of 5 to 10 less than in thermal ablation methods. Nevertheless, this power dissipation causes a non-negligible temperature rise which can be found most prominently around the tissue-electrode boundary.^8^ However, temperature itself is not the primary, nor the desired cell-killing mechanism.^9^ In fact, one of the most promising uses of IRE is to treat tumors, which are very close to major blood vessels, bile ducts (in liver), or nerves (in prostate), which limit thermal ablation methods like radiofrequency, laser or microwave ablation.^10^–^12^ The reason for this limitation is on one hand the risk of leaving residual vital tumor due to the heat sink effect induced from the cooling of the vessel. On the other hand, there is a significant risk of heat caused coagulation of sensitive structures like nerves and bile ducts with serious complications for the patient. However, the local electric field distribution, which is the most important factor for successful treatment with IRE is affected by the higher conductivity of blood and blood vessels.^13^–^15^ This means that additional care has to be taken when IRE is performed near blood vessels, where it is most preferred over the other thermal ablation techniques.

Currently, the manufacturer of the only certified medical device available for IRE treatments (NanoKnife, Angiodynamics, Latham, NY) recommends electrodes for ablation of liver tumors to be no more than 2.2 cm apart, positioned in parallel around the target volume. A total of 90 pulses with voltage to distance ratio of 1500–1700 V/cm, and 90 μs duration are recommended per electrode pair according to System Procedure Guide Software Version 2.2.0. The graphical user interface of the software provides a simple treatment planning option in two dimensions (2D), while not taking into account differences in electric conductivities of tissue between normal and tumor tissue.^16^

In this study we present a method for numerical patient-specific treatment planning for IRE, which takes into account the influence of blood vessels on electric field distribution. The method is illustrated on a 48-years-old. female patient with a recurrent metastasis directly adjacent to the last remaining hepatic vein after previous right-side hemi-hepatectomy and the successful treatment with IRE.

## Materials and methods

### Electric field computation and temperature distribution computation

Since IRE relies on applying local fields in excess of 600 V/cm in the whole target volume, electrodes need to be introduced in the target volume itself, but preferably minimizing the number of electrodes inserted in the tumor to preclude needle track metastasis seeding. The electric field is however affected by the local conductivity of tissue, which generally varies between different tissues, especially at frequencies, which are present in electroporation pulses (Table 1). Additionally, conductivity of tissue was shown to increase due to the electric field during the pulse delivery due to membrane electroporation.^17^,^18^ Together with the complex geometry of the target location (blood vessels, tumors and liver) this generally requires numerical computation of the electric fields.

In order to differentiate the tissues of different conductivities and separate the target volume from the healthy tissue a segmentation step is required in the treatment planning procedure.

We use a treatment planning procedure based on optimization in Matlab, while electric fields are solved in Comsol Multiphysics. The simulations consist of solving the Laplace equation for electric potential, given boundary conditions for electric potential on the electrodes. A stationary solver is used for the simulations, but iterated 6 times, increasing the conductivity of the tissues above electroporation thresholds between each iteration.^18^,^21^–^23^ From the electric field simulations, we obtained the electric field distribution and final volumes of tumor and liver covered with fields above the IRE threshold. Since more than one electrode pair is generally required to obtain clinically relevant treatment volumes in IRE, the electric field from each electrode pair are compared and the maximum value at each location is considered when evaluating the total coverage of the target volume.

For verifying the tissue heating during the treatment, the thermal dissipation of the electric field computation step can be used to set a heat source for a Pennes’ bioheat equation for a transient solver of temperature fields. To shorten simulation times, a duty-cycle approach is used, wherein we use the thermal dissipation multiplied by the duty cycle of the pulse delivery to model heating. This provides a comparable temperature increase in the bulk tissue^9^, but is numerically more stable and much faster. The reason for this is that it does not have to account for the very fast temperature rise during the 90 μs that the pulse is turned on in comparison to the 10000 times longer interval between pulses. All parameters of the electrical and thermal model were taken from the literature and are listed in Table 1.

### Patient data

**FIGURE 1.**
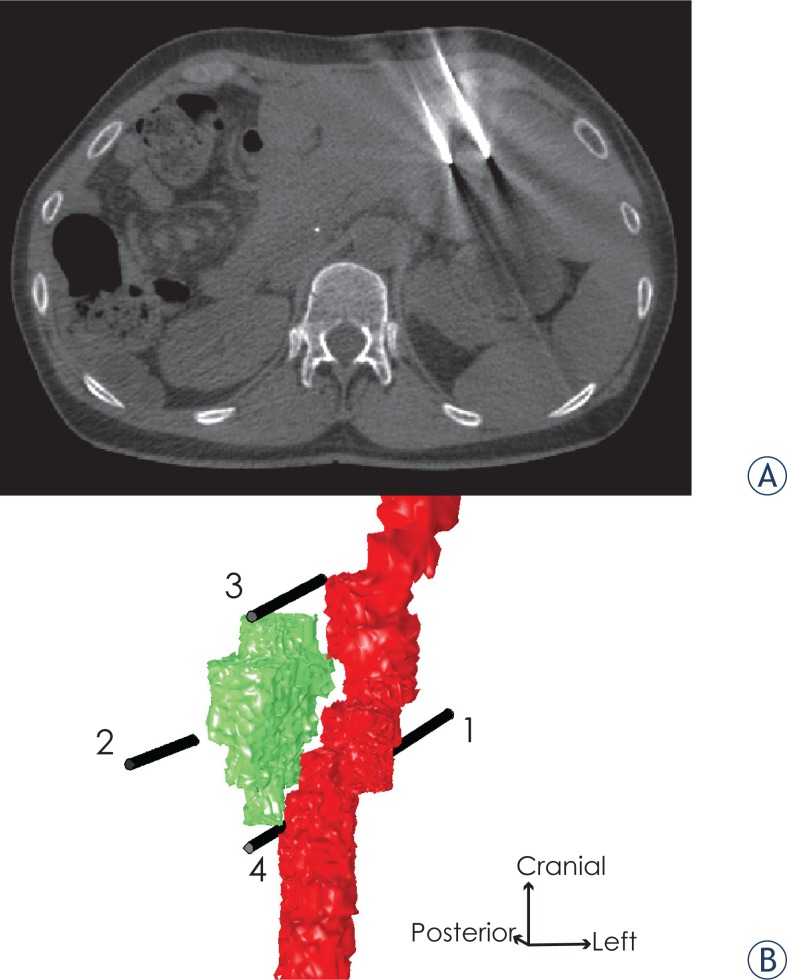
**(A)** CT image showing position of two of the electrodes (electrodes 1 and 2) inside the liver. **(B)** 3-D model showing the positioning of the electrodes in the model relative to the tumor (green) and the left liver vein (red). The liver, which was also included in the computational model is removed from the image for clarity, since it is completely encompassing the region of interest. The numbers identify each electrode.

The patient was a 48-years-old female who had previously undergone right hemi-hepatectomy for treatment of metastases of cholangiocellular carcinoma. During follow-up imaging a small (14 × 9 × 15 mm, i.e. 0.96 cm^3^) focal recurrent metastasis was detected in the remaining left liver. Since the metastasis was adjacent to the only remaining left hepatic vein it was not surgically resectable. Percutaneous CT guided IRE ablation was selected as the best treatment option to preserve this last liver vein since primary thermal ablations like radiofrequency ablation would have been contraindicated in this constellation. IRE can achieve complete ablation of tumors even nearby major blood vessels, since it is not negatively affected by their cooling effect such as thermal therapies^24^ where this so called heat sink effect may lead to incomplete tumor ablation. Furthermore, it is also commonly reported, that it is sparing for larger blood vessels^25^ which could have been damaged during thermal ablations. The patient was treated in the scope of the research project GoSmart (funded by the European Commission – grant agreement no. 600641). Ethical approval was obtained from Leipzig University Hospital Institutional Review Board under code AZ206 -13 – 15072013. Informed consent to use their personal data for scientific purposes was obtained from the patient. Treatment was performed using the above-mentioned pulse generator and the ablation protocol recommended from the manufacturer. Electrodes were positioned using CT guidance (Figure 1). Electrode exposure length for the treatment was 2 cm. All data was collected from clinical records or from the generator, where electric pulse data it is saved by default. The data is routinely recorded for improvement of quality, practice and performance of this novel treatment.

For the illustration case, we used MRI images of the patient acquired 3 weeks prior to the treatment. The images were anonymized and uploaded into the web based electric fields visualization tool Visifield (www.visifield.com, University of Ljubljana, Slovenia). Liver was segmented using automatic segmentation method for liver segmentation^26^ and the tumor and blood vessel were segmented manually. Interventional CT images obtained during the procedure were used to exactly reconstruct the electrode positions during treatment and to have these available for the numerical simulations. The reconstructed distances between the tips of the electrodes and angles between the tips of the electrodes are given in Table 2. It is also demonstrated, that the radiologist performing the procedure has managed to place the electrodes almost completely in parallel.

The pulse generator measures the pulse voltage and current during electric pulse delivery and stores the results in an xml document, which was parsed into Matlab. The same voltages, as were actually delivered during the actual treatment for each electrode pair were also used in a finite element computational model. The electric field distribution was computed only for the first pulse of each pulse train using a stationary solver, but taking into account increase of conductivity due to electroporation.

## Results

A total of 660 pulses were delivered in three sequences to six electrode pairs (pulses are always delivered to pairs of electrodes; sequentially, pairs of two electrodes are selected from available electrodes), with the electrode positioning and numbering as shown in Figure 1. Initially trains of 20 pulses were delivered (test pulses), then trains of 70 pulses were delivered, with some voltage adjustments (treatment pulses), and finally trains of 20 pulses were delivered (additional pulses) with lower voltages. Table 3 lists all delivered pulses and pulse parameters.

**FIGURE 2.**
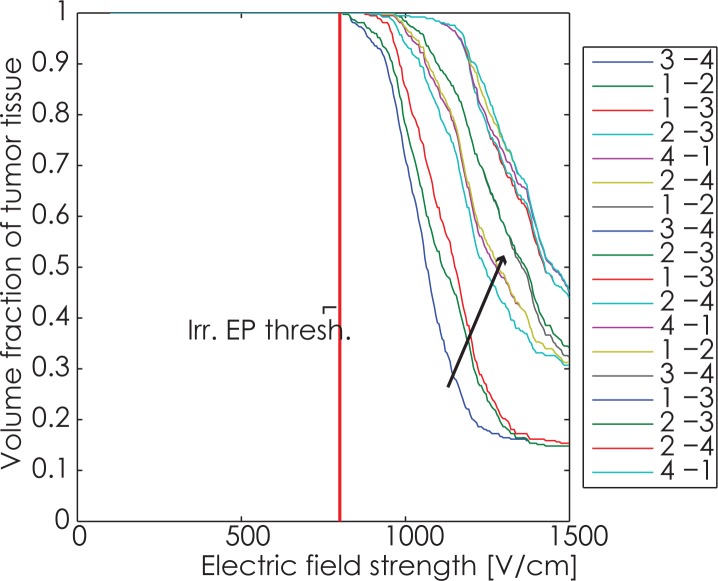
Coverage progression after delivery of pulses to each electrode pair. The graph shows the combined maximum fields in the tumor following each electrode pair. Electrode pair progression is the same as in Table 3. Arrow indicates the direction of increase of coverage with delivery of successive electric pulses. The graph shows that the first electrode pair already covers the whole tumor with electric fields above the irreversible electroporation threshold.

**FIGURE 3.**
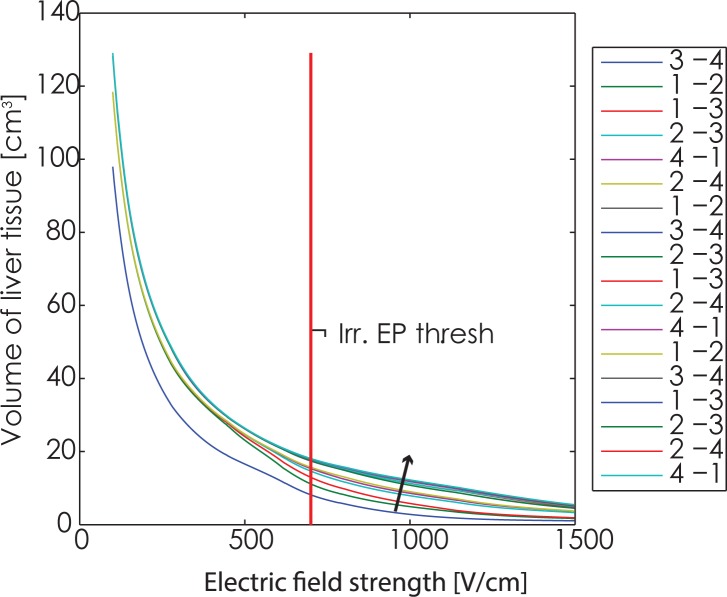
Electric field coverage in the liver tissue. The tumor tissue is not included in the volume on this graph. The graph shows the combined maximum fields in the liver following each electrode pair. Electrode pair progression is the same as in Table 3. Arrow indicates the direction of increase of coverage with delivery of successive electric pulses.

The root mean square error (RMSE) of the computed currents versus measured currents from the pulse generator is 3.8 A. The cumulative coverage of tumor with electric fields after each electrode pair is shown in Figure 2, while the coverage of liver tissue is shown in Figure 3. While the IRE threshold for tumors has not yet been rigorously determined, we are using a value of 800 V/cm in the following graphs, consistent with previous work.^6^,^23^,^27^ For liver we used IRE threshold determined from experiments on rabbit livers – 700 V/cm.^21^ To be noted however, this value is for pulse trains of 8 pulses. It is very likely that actual IRE thresholds are much lower.^7^,^28^

In the post-IRE contrast enhanced CT images, a hypodense region can be seen in the area where the treatment was performed. We used this hypodense region to estimate the total IRE lesion as was previously suggested.^29^ The volume of this hypodense lesion was 20.03 cm^3^. From Figure 3, the volume of the IRE lesion in the liver is 18.4 cm^3^. Together with the tumor (0.57 cm^3^) in the simulations, the total volume of the computed lesion equals 19.9 cm^3^, which is comparable to the lesion size determined by CT.

**FIGURE 4.**
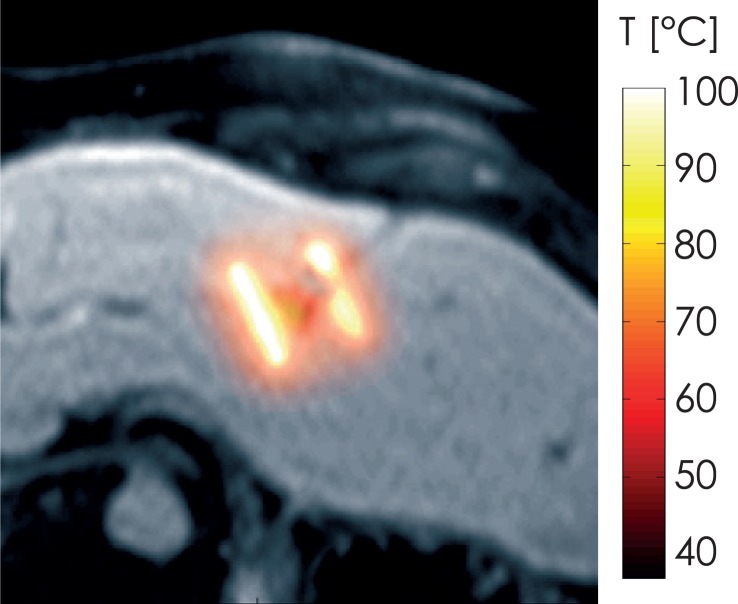
One slice showing computed temperature distribution after all pulses from the 7^st^ pulse train (electrode pair 1 – 2 at 3000 V) superimposed on the corresponding MRI slice of the model.

**FIGURE 5.**
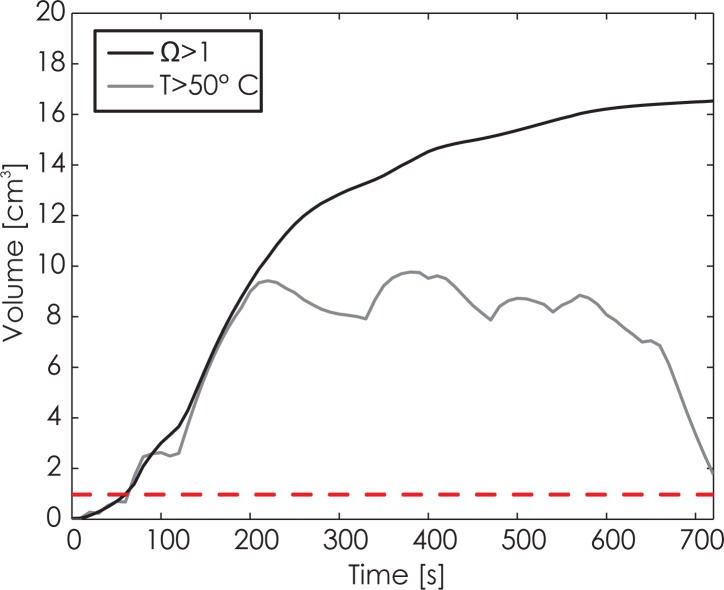
Graph of the total volume of tissue with Arrhenius integral above 1, indicating a high probability of thermal damage. Second graph shows the volume of tissue above 50°C, which is consistent with the volume of tissue around the electrodes.

Figure 4 shows a single slice of temperature data after the 7^th^ pulse treatment set (first train of the treatment pulses, and the train with highest pulse amplitude). The tumor is heated slightly more than the surrounding tissue, as its perfusion is lower, and also the conductivity is higher than that of the liver tissue, which both contributes to a higher Joule heating. Temperatures above 50 C are typically used for indication of thermal tissue damage.^1^ Therefore, we also show the volume of tissue above this threshold in Figure 5. Interesting to note is, that temperatures above 70°C are located less than 4 mm from the electrodes. If we approximate this volume with four cylinders, each with a radius of 4 mm and height of 28 mm (length of active electrode region with 4 mm added on either side), the total volume of high temperature caused by the electrodes is 5.6 cm^3^, which is consistent with the volume of tissue above 50°C shown in Figure 5.

The curve in Figure 5 shows, that the 63% probability of thermal damage, determined by the Arrhenius rate equation, increases strongly in the first 250 s while later the slope levels off. This is caused by the fast increase in temperature around the electrodes, and then a slower increase in temperature in the more distant areas. Finally, the slope gets flatter, since the heating does not extend further from the electroporated area, the pulse amplitudes start to decrease, and diffusion moves the heat into tissue further away.

## Discussion

The presented numerical results and clinical follow-up show that IRE is efficient at treating tumors in the immediate vicinity of major blood vessels. Since IRE is unaffected by the cooling of blood vessels, it is supposed to be not limited by their vicinity. The results of our computational model show a good correlation between the modelled IRE, electrical measurements during treatment, and imaging results. The tumor treatment has been classified as a complete ablation, and has shown no recurrence in the 6 months follow-up.

With the number, amplitude, and duration of pulses in the presented treatment, it is therefore clear, that a non-negligible temperature rise occurs. Since IRE has also been classified as a non-thermal tissue ablation technique in the literature^24^,^30^, it needs to be clarified, that non-thermal does not indicate that there is no temperature rise, but rather, that the temperature is not the main mechanism which induces cell death. Our model clearly shows that some areas of the lesion do heat up significantly (Figure 5), and the temperature rise is also consistent with experimental results from the literature.^31^ Although our results show, that IRE is not an exclusively nonthermal treatment, *i.e.* there is significant heating present in the vicinity of electrodes, the total treatment volume is significantly higher than the volume based on thermal effects would be expected. A limitation of the model is, that we assumed that pulses were delivered constantly at 1 Hz, while in reality, the pulses were delivered in synchronization with the patient’s ECG, which can realistically be up to 100 beats per minute, and would consequently result in a higher temperature rise. In Figure 4, some areas in immediate vicinity of the electrodes are heated to temperatures of more than 100°C, because there was no term for boiling included in the numerical model. In fact, these high temperatures could indicate that some tissue boiling actually occurs near the electrode tips. This could explain the gas bubbles visible in the post-treatment CT images, and which are consistent with reports in the literature.^32^ Another reason for these findings could be a gas formation due to electrolysis.^33^ However, the temperature drops to below 70°C approximately 4 mm away from the electrodes in this specific case.

The coverage of the target tumor with electric fields was very high. The IRE threshold electric field, which depends on the type of number, duration of pulses, and tissue types of liver tumors has not been firmly established yet.^6^ In this work, we assumed a value of 800 V/cm for tumor tissue. Nevertheless it can be seen in Figure 2, that almost the whole tumor is covered by electric field of this strength already in the first two pulse trains between electrode pairs 3–4 and 1–2. That, and the very high temperatures achieved in the model seem to indicate, that the used voltages and pulse numbers^34^ were considerably higher than necessary to achieve complete treatment of the tumor.

When liver tumors are surgically treated, at least a safety margin of 0.5 to 1 cm of liver tissue around the tumor is resected to ensure removal of any micrometastases surrounding the tumor and thereby to prevent local tumor recurrence. For the same reason there is a need to achieve a similar safety margin around the tumor in IRE treatments as well, and the IRE lesion in the presented case is larger than this goal. Assuming an elliptical approximation of the tumor, the volume of an ellipsoid with the semi-axes increased by 1 cm relative to the tumor, the required lesion volume would be 18.05 cm^3^. This corresponds also to the presented case and should be accounted for every treatment planning.

## Conclusions

Treatment planning using accurate computer models was recognized as important for electrochemotherapy and irreversible electroporation.^2^,^9^,^23^,^35^,^36^ On the one hand appropriate numerical treatment planning assures sufficient coverage of the clinical target volume with electric field sufficiently high for efficient tumor treatment also in the vicinity of blood vessles^15^ and thereby to prevent local recurrences. On the other hand it enables the prediction and control of temperature thus avoiding thermal tissue damage in critical structures, such as nerves or bile ducts. Regarding the strength of the electric field in the presented case, significant overtreatment can be assumed, since electric fields in the tumor were higher than 900 V/cm. In the future monitoring of electric field in real time^37^ and fast near-real time treatment planning will improve the adjustment of the electric pulse parameters. This should allow to preserve the efficiency and reliability of the treatment by avoiding heat induced adverse events. More precise planning will also enable the treatment of larger targets, while limiting thermal damage.^38^ An important finding of this study was, that the most heating occurs at the surface of the electrodes. This fact should instruct the clinical user applying the manufacturer recommended voltage settings of the NanoKnife system to avoid placement of the electrodes at a distance of less than 4 mm from at-risk structures in comparable cases in order to avoid thermal damage to these structures.

## Figures and Tables

**TABLE 1.** Parameters of the electrical and thermal model

**Property**	**Value**	**Reference**
σ_L_ — Liver initial conductivity	0.091 S/m	Haemmerich *et al.*^10^
σ_L_ — Liver final conductivity	0.45 S/m	Cukjati^17^
σ_T_ — Tumor initial conductivity	0.4 S/m	Haemmerich *et al.*^16^
σ_T_ — Tumor final conductivity	1.6 S/m	Extrapolated from Cukjati^17^
σ_Vfinal_ — Vessel initial conductivity	0.7 S/m	Marčan *et al.*^15^
σ_Vfinal_ — Vessel final conductivity	1.05 S/m	Marčan *et al.*^15^
αT — Tissue conductivity thermal coefficient	1.5 %/K	Haemmerich *et al.*^16^
C_T_ — Tissue thermal capacity	3540 J/(kg K)	Hasgall *et al.*^19^
ρ_T_ — Tissue density	1079 kg/m^3^	Hasgall *et al.*^19^
k_T_ — Tissue thermal conductivity	0.52 W/(m K)	Hasgall *et al.*^19^
ω_b_ —Blood perfusion	1.8 mL /s /100 mL	Hasgall *et al.*^19^
C_b_ —Blood thermal capacity	3840 J/(kg K)	Hasgall *et al.*^19^
ρ_B_ — Blood density	1060 kg/m^3^	Garcia *et al.*^9^
T — Initial tissue temperature	310 K	
q’’’ — Tissue metabolic heat generation	10740 W/m^3^	Hasgall *et al.*^19^
E_a_ — Activation energy	5.06×10^5^ J/mol	Henriques *et al.*^20^
ζ — Frequency factor	2.984×10^80^ s^−1^	Henriques *et al.*^20^
R — Universal gas constant	8.314 J/(mol*K)	
σ_L_ — Electrode conductivity	10^6^ S/m	
k_E_ — Electrode thermal conductivity	15 W/(m K)	Garcia *et al.*^9^
ρ_E_ — Electrode density	6000 kg/m^3^	Garcia *et al.*^9^
C_E_ — Electrode thermal capacity	500 J/(kg K)	Garcia *et al.*^9^

**TABLE 2.** Reconstructed distances and angles between the electrodes

**Electrode pair**	**Distance [mm]**	**Angle [°]**
1 — 2	18	4.1
1 — 3	14	1.2
1 — 4	12	1.8
2 — 3	15	3.2
2 — 4	12	5.2
3 — 4	17	2.0

**TABLE 3.** List of delivered pulses and comparison with computed currents

**Pulse sequence**	**Pulse train**	**Electrode pair**	**Voltage [V]**	**No. of pulses**	**Measured current [A]**	**Computed current [A]**	**Error [%]**
*Test pulses*	1	[3,4]	2720	20	26.6	27.4	3
	2	[1,2]	2550	20	21.5	23.9	11
	3	[1,3]	2380	20	23.6	25.8	9
	4	[2,3]	2210	20	21.3	21.0	−2
	5	[4,1]	2200	20	22.5	25.7	14
	6	[2,4]	1650	20	17.4	16.2	−7
*Treatment pulses*	7	[1,2]	3000	70	30.5	28.4	−7
	8	[3,4]	2720	70	30.5	27.4	−10
	9	[2,3]	2405	70	30.3	22.8	−25
	10	[1,3]	2380	70	30.7	25.9	−16
	11	[2,4]	2200	70	31.1	22.3	−28
	12	[4,1]	2200	70	29.3	25.6	−13
*Additional pulses*	13	[1,2]	2380	20	24.2	22.1	−9
	14	[3,4]	2380	20	28.5	23.6	−17
	15	[1,3]	1960	20	22.1	20.6	−7
	16	[2,3]	1820	20	20.5	16.8	−18
	17	[2,4]	1540	20	18.9	14.9	−21
	18	[4,1]	1540	20	17.2	17.1	0
